# 

**Published:** 1924-06

**Authors:** 


### HOSPITAL and HEALTH REVIEW

*Established 1886.* *No. 1875. Vol. LXXIII*

*New Series.* Vol. III. No. 33. *June,* 1924. Price Sixpence

CONTENTS.

| | PAGE |
|---|---|
| The Finger of the State | 163 |
| International Health | 164 |
| Notes and Comments:— Grandiose but Impracticable—The British Hospitals Association—Certification and Criminal Responsibility—Hospital Sunday—The Commission on Health Insurance—How to Arrest a Hiccough—The Quest of the Complexion—Health by Wireless—An Echo of 1871—The Care of the Convalescent—Sun Baths and Shaving—The School Doctor—Pensions for Nurses—The Case of the Child Offender | 165 |
| Hospital Men of Mark: Mr. J. Courtney Buchanan, C.B.E. | 169 |
| Change and Monotony in the Factory | 170 |
| Can the Voluntary System Continue? | 171 |
| The Medical Mission Field | 173 |
| Reducing Venereal Disease in Belgium | 173 |
| Please! | 173 |
| Health and Comfort at Wembley. By Theodore Ruete | 174 |
| Hospital Progress and Finance | 175 |
| The Hospital Almoner. By Miss M. W. Edmindson | 177 |
| Hospital Officers Conference | 178 |
| Enham Village Centre | 179 |
| Chinese Foot Binding | 179 |
| How Glands Influence Character: A New Conception in Physiology | 180 |
| League of Remembrance | 181 |
| Contributory Benefactors | 181 |
| An East End Hospital | 182 |
| Correspondence | 182 |
| Accounting in Relation to Hospital Administration. By Joseph E. Stone, F.S.A.A., F.S.S. | 183 |
| Royal Society Conversazione | 185 |
| The Hospital, Nursing, and Midwifery Exhibition | 185 |
| A Private Patients' Hospital | 185 |
| Reviews of Books | 186 |
| Echoes of the Month | 189 |
| Hospital and Health News | 190 |
| Points from Hospital Reports | 191 |
| Answers to Correspondents | 192 |
| Recent Appointments | 192 |

